# The role of perceived quality of care on outpatient visits to health centers in two rural districts of northeast Ethiopia: a community-based, cross-sectional study

**DOI:** 10.1186/s12913-024-11091-z

**Published:** 2024-05-10

**Authors:** Mohammed Hussien

**Affiliations:** Department of Health Systems Management and Health Economics, School of Public Health, College of Medicine and Health Sciences, Bahir Dar University, Bahir Dar, Ethiopia

**Keywords:** Quality of care, Outpatient visits, Health centers, Ethiopia

## Abstract

**Background:**

Patients who have had a negative experience with the health care delivery bypass primary healthcare facilities and instead seek care in hospitals. There is a dearth of evidence on the role of users’ perceptions of the quality of care on outpatient visits to primary care facilities. This study aimed to examine the relationship between perceived quality of care and the number of outpatient visits to nearby health centers.

**Methods:**

A community-based cross-sectional study was conducted in two rural districts of northeast Ethiopia among 1081 randomly selected rural households that had visited the outpatient units of a nearby health center at least once in the previous 12 months. Data were collected using an interviewer-administered questionnaire via an electronic data collection platform. A multivariable analysis was performed using zero-truncated negative binomial regression model to determine the association between variables. The degree of association was assessed using the incidence rate ratio, and statistical significance was determined at a 95% confidence interval.

**Results:**

A typical household makes roughly four outpatient visits to a nearby health center, with an annual per capita visit of 0.99. The mean perceived quality of care was 6.28 on a scale of 0–10 (SD = 1.05). The multivariable analysis revealed that perceived quality of care is strongly associated with the number of outpatient visits (IRR = 1.257; 95% CI: 1.094 to 1.374). In particular, a significant association was found for the dimensions of provider communication (IRR = 1.052; 95% CI: 1.012, 1.095), information provision (IRR = 1.088; 95% CI: 1.058, 1.120), and access to care (IRR = 1.058, 95% CI: 1.026, 1.091).

**Conclusions:**

Service users’ perceptions of the quality of care promote outpatient visits to primary healthcare facilities. Effective provider communication, information provision, and access to care quality dimensions are especially important in this regard. Concerted efforts are required to improve the quality of care that relies on service users’ perceptions, with a special emphasis on improving health care providers’ communication skills and removing facility-level access barriers.

**Supplementary Information:**

The online version contains supplementary material available at 10.1186/s12913-024-11091-z.

## Background

Essential health service coverage, which is one of the two dimensions of universal health coverage, is also an indicator of progress towards sustainable development goals. The goal of the service coverage dimension of universal health coverage is that people in need of health services receive them and that the services received are of sufficient quality to achieve potential health gains [1].

Despite various efforts put in place as part of the main global agenda to facilitate access and effective coverage, persistent inequalities in accessing and using essential health services exist both between and within countries [2]. A recent global estimate showed that the excess deaths of 3·6 million people in low- and middle-income countries were due to the non-utilization of health care [3]. Essential health service coverage involves receiving a wide range of promotive, preventative, curative, rehabilitative, and palliative health services [2]. Outpatient visits with primary care providers are for many people the most frequent contact with health services, and often provide an entry point for subsequent health care [4]. Outpatient service use, which is measured by the number of outpatient visits per person per year, is one of the proposed core indicators for health care delivery [5]. The use of outpatient services can be used as a proxy for essential services coverage and portray the image of the health care system. Low rates of outpatient visits are suggestive of limited access and low quality of care [6].

There has been an increasing emphasis on the importance of improving health-care quality as a critical component of the path to universal health coverage, along with expanding service coverage and financial risk protection [7–9]. Low-quality health services, despite their availability, are a major deterrent to achieving effective universal health coverage. This is due to the fact that communities will not use services that they distrust and are of little benefit to them [7]. In line with the global trend, the health system in Ethiopia has shifted its focus from increasing coverage of essential health services to quality improvement. Parallel to expanding access to services, the Ministry of Health identified five priority areas in its strategic plan that require radical shifts, one of which is transformation in quality of care [10].

Quality of health care is a broad concept that has been assessed using various measurement approaches in order to better understand it [11]. The emphasis on measuring healthcare quality has shifted away from the perspectives of healthcare providers towards people-centered approaches that rely on user perceptions [12]. Patients’ perceptions of health-care quality, which are based on a combination of patient experiences, rumor, and processed information, are becoming an important component of quality measurement because they are significant drivers of healthcare utilization [11].

The literature supports the view that a positive experience with healthcare services would prompt patients to revisit the healthcare facility [13–15] and attend for scheduled appointments [16]. This is based on the view that patients with negative health-care delivery experiences will lose trust in service providers, and they will be less likely to use services once more [17, 18]. It was also documented that a greater number of problems related to the quality of primary care provisions, as perceived by users, discourages the use of health care [19]. The perception of the quality of care also has an effect on the choice of healthcare facility. Evidence indicated that patients who had little faith in primary health care facilities sought treatment elsewhere, preferably in hospitals [20]. Service users with a positive experience with care delivery also recommend the service provider to others. A nationally representative survey conducted in 14 low-income, middle-income, and high-income countries showed that high ratings of user-reported quality of care is a positive predictor of patients’ recommendations of the healthcare facility to a friend or family member [21].

The quality of healthcare must be assessed and improved on a regular basis to foster optimum health care utilization and health outcomes. A recent study in Ethiopia investigated the perceived quality of medical services at public hospital outpatient units [22]. However, the quality of outpatient services in primary health care facilities is not well addressed from the perspective of the service users. Furthermore, while some studies looked at the effect of perceived quality of care on behavioral intentions [13–15] and the choice of health facility levels [20, 23], there is little scientific evidence on the relationship between perceived quality of care in primary care facilities and the frequency of outpatient visits in the same facility. Therefore, the purpose of the current study was to examine the association between service users’ perceptions of the quality of care and the number of outpatient visits to nearby health centers among households in two rural districts of Ethiopia.

Improving the quality of health care is among the top priorities of Ethiopia in its health sector strategic plan [10]. The findings of this study will inform health authorities, service providers, and other relevant actors on the role of service users’ perceptions of the quality of care in their choice of health facility, which can be a proxy for the performance of the larger health system. It will also provide useful information to identify areas of focus that require the attention of relevant stakeholders striving to improve the quality of care.

## Methods

### Study design and setting

A community-based cross-sectional study was conducted in rural parts of two neighboring districts in northeast Ethiopia, Kalu and Tehulederie. Kalu has nine health centers serving a population of around 235,000, of which 89% live in rural areas. In Tehulederie, there are five health centers and one primary hospital designated to provide services for a population of more than 145,000, of which 88% are rural dwellers [24].

In Ethiopia, health services are provided by a network of health facilities arranged in a three-tier health care delivery model: primary healthcare units, general hospitals, and specialized hospitals. A primary healthcare unit consists of health posts, health centers, and primary hospitals. A health center is attached to five satellite health posts to provide both preventive and curative services to approximately 25,000 people, while a health post delivers preventive, promotional, and selected curative interventions at the community level. Health centers are assumed to be the first level of outpatient service delivery points in the three-tier system. Primary and general hospitals both offer inpatient and outpatient services, but to varying degrees. The third-tier system includes a specialized hospital dedicated to providing tertiary-level health care. The population is free to choose between health care facilities without being constrained by a gatekeeping policy; however, patients are encouraged to use the lower-level health facility first before proceeding to the next higher level via upward referral [10].

### Sample size and sampling

The data used in this study comes from a research project examining the sustainability of a community health insurance (CHI) scheme in Ethiopia. As part of this project, a sample size of 1257 was calculated for a companion article [25], of which 1081 eligible households took part and provided complete data relevant to the current study. The study population of interest consisted of rural households that had been enrolled in the CHI scheme. This includes households that were active members at the time of the study and those that dropped out of the scheme. Households that had not visited health centers for outpatient services in the 12 months period prior to the study were excluded to minimize recall bias in measuring the perceived quality of care.

A three-level multistage sampling was used to recruit study participants. First, 12 clusters of *Kebeles* organized around a health center catchment area were selected. Then, 14 rural *Kebeles* were drawn at random proportional to the number of *Kebeles* under each cluster. Accordingly, nine *Kebeles* from Kalu and five from Tehulederie were included. A list of households that have ever been enrolled in the CHI was obtained from each Kebele’s membership registration logbook. Using random number generator software, the required sample was generated randomly from each *Kebele*, proportional to the number of households that have been enrolled in the scheme.

### Data collection and variables

The data were collected from February 4 to March 21, 2021, through face-to-face interviews with household heads at their homes using a structured questionnaire via an electronic data collection platform. Data was collected on characteristics of the household head, including age, gender, current marital status, and educational attainment. Data was also collected on place of residence, family size, economic status, household CHI membership status, presence of chronic illness in the household, perceived health status of the household, perception of the quality of health care received from the nearby health center, and number of outpatient visits to the nearby health center by any member of the household (see Supplementary file). The data collectors submitted the completed forms to a data aggregating server on a daily basis, allowing us to review them and simplify the supervision process. Health extension workers assisted data collectors in tracking the sampled households because they are primarily responsible for providing home-based health services in rural areas and are familiar with each household’s location.

The outcome variable of interest is the number of outpatient visits to nearby health centers. It refers to a household’s outpatient trips to a nearby health center for curative health care in the year preceding the study. It is a count data with all observations greater than zero because households that had not used health care in the previous 12 months prior to data collection were excluded from the study. This was done to reduce recall bias on some of the items designed to measure perceived quality of care. Per capita outpatient visit, which is the average number of outpatient visits to nearby health center made by a household member during the one-year period preceding the study, was also calculated to allow comparison across covariates.

The number of outpatient visits was assumed to be influenced by the perceived quality of care and other household characteristics, which were included as covariates. The perceived quality of care, which is the main independent variable of interest, was assessed using a 17-item scale developed following a thorough review of validated tools for outpatient visit encounters in low- and middle-income settings including Ethiopia [26–30]. Respondents were asked to rate how much they agreed on a set of items relating to their experiences with health services received in the outpatient departments of a nearby health center, which is thought to be the usual source of health care. Each item was designed with a 5-point response format, with 1 representing strongly disagree, 2 disagree, 3 neutral, 4 agree, and 5 strongly agree.

To allow for comparisons of summary scores of overall perceived quality of care, quality dimensions, and measurement items on a common scale, the 5-point response was converted to scores of 0, 2.5, 5.0, 7.5 and 10 respectively, and mean scores were arithmetically transformed to a continuous scale of 0 to 10 [31, 32]. A mean score of the overall perceived quality of care was calculated from the total items and was handled as a continuous variable. The scores for the 17 items were translated into five quality dimensions using exploratory factor analysis. A mean score is also computed for each dimension based on the items that load in that dimension.

The covariates in this study are based on Anderson’s behavioral model of health service use, which contends that people’s use of health services is driven by their predisposition, enabling factors to access services, and their needs for care [33]. Based on this framework, the following characteristics were considered to control for potential confounding factors in the association between perceived quality of care and choice of health facility for outpatient visits: predisposing characteristics (age, gender, marital status, educational attainment, place of residence, and household size); enabling factors (wealth index and health insurance coverage); and the need for care (chronic illness and perceived health status).

The wealth index was created using the principal component analysis method. The scores for 15 different types of assets were converted into latent factors, and a wealth index was generated using the first factor that explained most of the variations. Based on the index, the study households were categorized into three wealth tertiles: poor, middle, and rich. Perceived health status was rated as poor, moderate, or good based on a household head’s subjective assessment of the household’s health status.

The questionnaire was pre-tested on 84 randomly selected participants prior to data collection. A cognitive interview on selected items was conducted as part of the pre-test with eight respondents using the verbal probe method to determine whether the items and response categories were well understood and interpreted by the potential respondents. As a result, six quality measurement items were removed and the wording of some items was modified on the translated local language.

### Data analysis

Stata Statistical Software, release 17 was used to analyze the data. The validity of the measurement scale on perceived quality of care was assessed using exploratory factor analysis. The details on the factor analysis procedures and its results are thoroughly described in another companion article [34].

Since the outcome variable of interest is a count data, that is the annual number of outpatient visits made by the household, Poisson regression was considered as a standard analysis model. Because the number of outpatient visits is a count response variable with all observations greater than zero, the analysis would employ either zero-truncated Poisson regression (ZTP) or zero-truncated negative binomial (ZTNB) regression models. Poisson model assumes that the variance is equal to the mean. A test of goodness of fit was performed and it showed an overdispersion (the variance of outpatient visits was more than twice its mean). The Negative Binomial model is appropriate when the dependent variable is over-dispersed [35]. In addition, the Akaike information criterion (AIC), Bayesian information criterion (BIC) and Deviance Information Criterion (DIC) statistics were computed to select the best fitted model. Accordingly, the values of the AIC, BIC and DIC statistics of the zero-truncated negative binomial model were substantially lower than those of the zero-truncated Poisson model, indicating a better fit to run the multivariable regression analysis.

The basic Poisson model is given by a regression equation of the form [36],

1 $${\rm{ln}}\left( r \right) = {\beta _0} + {\beta _1}{X_1} + {\beta _2}{X_2} + \cdots + {\beta _i}{X_i}, \ldots ,$$

where β_0_ is the intercept, β_1_, β_2_,. . β_i_ are the Poisson regression coefficients of *i* explanatory variables whose values are at *X*_*1*_, *X*_*2*_*…, X*_*i*_, and, *r* is the incidence rate.

When interpreting results, it is preferable to use the incidence rate ratio (IRR) rather than the regression coefficients to investigate the effect of predictor variables on the count response variable. By taking the exponent of the coefficient, we obtain the *incidence rate ratio* (IRR) as follows,

2 $$r = {e^{({\beta _0} + {\beta _1}{X_1} + {\beta _2}{X_2} + \cdots + {\beta _i}{X_i})}}, \ldots ,$$

The estimated IRR for the individual covariate ${x}_{j}$ is defined as:

3 $$IR{R_j} = {e^{{{\hat \beta }_j}}}, \ldots ,$$

where ${\widehat{\beta }}_{j}$ is the *j*^*th*^ estimated regression coefficient.

After adjusting for the confounding effect of covariates, the measures of association estimated the association between the perceived quality of care and annual number of outpatient visits. The existence of a statistically significant association was determined at *p*-values of < 0.05. The degree of the association was assessed using incidence rate ratio (IRR), and their statistical significance was determined at a 95% confidence interval. In the multivariable regression analysis, two models were estimated. Model I demonstrated the association between overall perceived quality of care and the number of outpatient visits, whereas Model II showed the link between the dimensions of perceived quality of care and the number of outpatient visits, both after controlling for covariates.

## Results

### Background characteristics of the study participants

The study included 1081 participants who had visited a health center at least once in the previous 12 months. The study participants’ average age was 49.25 years, with slightly more than half (51.3%) between the ages of 45 and 64, and 12.7% being 65 and older. among the total study participants, 938 (86.8%) were male, and 1003 (92.8%) were currently married. One-fifth of the study participants (20.9%) had a formal education, and 62.7% had a household size of five or above.

Nearly nine out of ten households (87.1%) were active members of the CHI scheme at the time of the study. A quarter of households (25.7%) had one or more individuals with a known chronic illness who had been informed by a healthcare provider. One-third of respondents (33.6%) rated their household health status as good, while 511 (47.3%) and 207 (19.1%) rated it as moderate and poor, respectively (Table 1).

**Table 1:** Per-capita outpatient visits compared across respondent characteristics

Variables	Categories	Total (*n* = 1081)	Percent	Per-capita OPD visits		F- test/t- test
				M	SD	
Age in years	25–44	389	36.0	0.77	0.58	32.22**
	45–64	555	51.3	0.99	1.06	
	65+	137	12.7	1.58	1.61	
Gender	Male	938	86.8	0.93	0.93	-4.55**
	Female	143	13.2	1.35	1.43	
Current marital status	Unmarried	78	7.2	2.09	2.22	10.15**
	Married	1003	92.8	0.90	0.83	
Attend formal education	No	855	79.1	1.04	1.14	3.61**
	Yes	226	20.9	0.77	0.45	
Place of residence	Rural	622	57.5	1.13	1.17	5.39**
	Semi-urban	459	42.5	0.79	0.79	
Household size	< Five	403	37.3	1.43	1.46	11.45**
	≥ Five	678	62.7	0.72	0.53	
Wealth tertile	Poor	361	33.4	1.28	1.35	23.93**
	Middle	360	33.3	0.91	1.00	
	Rich	360	33.3	0.77	0.56	
Current insurance status	Ex-member	139	12.9	0.76	0.64	-2.80*
	Active member	942	87.1	1.02	1.08	
Chronic illness	No	803	74.3	0.81	0.71	-9.87**
	Yes	278	25.7	1.50	1.55	
Perceived health status	Poor	207	19.1	1.68	1.69	63.68**
	Moderate	511	47.3	0.86	0.63	
	Good	363	33.6	0.77	0.85	

*P < 0.01, **p < 0.001

OPD: Outpatient department

### Perceptions of the quality of care

The exploratory factor analysis extracted five dimensions of quality of care: technical care, patient-provider communication, information provision, access to care, and trust in care providers. On a scale of 0–10, the mean score of the overall perceived quality of care was 6.28 (SD = 1.05). Provider communication had the highest mean score (M = 7.23, SD = 1.27) of the five quality dimensions, while information provision had the lowest score (M = 5.58, SD = 1.73). The mean score of the quality dimensions and each measurement item is displayed by Table 2.

**Table 2:** Mean score of perceived quality of care, its dimensions and measurement items (0–10 scale)

Factors and items	Mean	SD	95% CI
**Technical care**	**6.04**	**1.91**	**5.93, 6.16**
The necessary laboratory tests were performed	6.15	2.30	6.01, 6.29
Health care providers perform the necessary physical examinations	6.03	2.36	5.89, 6.17
Health care providers make good diagnoses	5.95	2.21	5.82, 6.08
**Patient-provider communication**	**7.23**	**1.27**	**7.15, 7.30**
Health care providers actively ask questions to understand your situation	7.55	1.46	7.46, 7.64
Health care providers listened to you carefully what you had to say	7.45	1.37	7.37, 7.53
Health care providers treated you with courtesy and respect	6.69	2.09	6.56, 6.81
**Information provision**	**5.58**	**1.73**	**5.48, 5.69**
Health care providers clearly explained the use and side effects of medicines	5.36	2.48	5.21, 5.51
Health care providers clearly explained the results of tests and examination	5.31	2.43	5.17, 5.46
Health care providers explain things in a way you could understand	6.17	2.18	6.04, 6.30
Health care providers spent sufficient time examining patients	5.49	2.52	5.34, 5.64
**Access to care**	**6.18**	**1.47**	**6.10, 6.27**
Patients do not wait long in the health center to receive treatment	5.37	2.52	5.22, 5.52
All prescribed medicines are available on the spot	5.44	2.55	5.29, 5.59
Health center assistants are friendly and helpful to patients	6.67	2.01	6.55, 6.79
The health center serves all patients fairly	7.26	1.99	7.14, 7.38
**Trust in care providers**	**6.65**	**1.38**	**6.57, 6.73**
Treatment is effective for recovery and cure	6.56	1.85	6.45, 6.67
Health care providers prescribe appropriate medicines for patients	6.93	1.61	6.84, 7.03
You have confidence in the competence of health care providers	6.46	1.79	6.35, 6.57
**Overall perceived quality of care**	**6.28**	**1.05**	**6.22, 6.35**

SD: Standard Deviation; CI: Confidence Level

### Frequency of annual outpatient visits

**Fig. 1:**
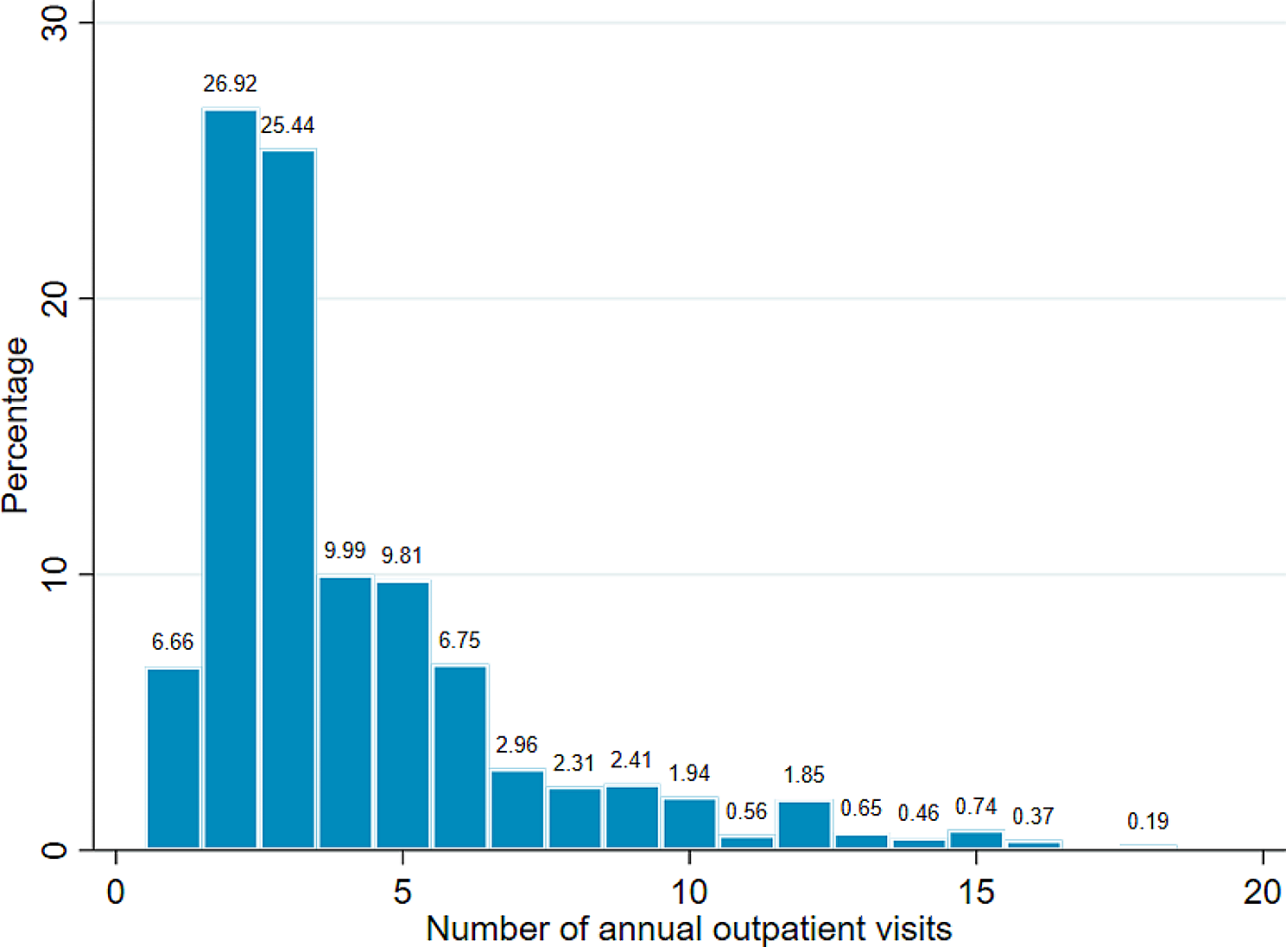
Frequency distribution of outpatient visits (number of observations = 1081)

Frequency distribution of outpatient visits showed that more than half of the study households (52.4%) had two or three outpatient visits per year, with other counts having a smaller percentage. The maximum distribution of outpatient visits was 18 visits (0.2%) over one year. A typical household makes roughly four outpatient visits to health centers per year. The variance of outpatient visits was 8.47, which was slightly more than twice the mean of 4.10, indicating data overdispersion. Figure 1 depicts the frequency distribution of outpatient visits. Health-care utilization as measured by the number of outpatient visits per household member was 0.99 visits per person per year. Table 1 presents the per capita outpatient visits across different respondent characteristics.

### Multivariable analysis using zero-truncated negative binomial regression model

The results of the multivariable zero-truncated negative binomial regression are presented in Table 3. In model I, the overall perceived quality of care was included in the regression analysis after adjusting for the confounding effect of the covariates. Accordingly, a positive perception of the quality of care is significantly associated with an increase in the annual number of outpatient visits. As the mean score of perceived quality of care increased by one unit, the number of outpatient visits to a nearby health center increased by 25.7% (95% CI: 1.210, 1.306; *p* < 0.001).

**Table 3:** Multivariable analysis using zero-truncated negative binomial regression model on predictors of outpatient visits to health centers

Explanatory variables	Model I			Model II	
	IRR (95% CI)	*p*-value		IRR (95% CI)	*p*-value
Technical care				1.021 (0.995, 1.048)	0.119
Provider communication				1.052 (1.012, 1.095)	0.011
Information provision				1.088 (1.058, 1.120)	< 0.000
Access to care				1.058 (1.026, 1.091)	< 0.000
Trust in care providers				1.019 (0.987, 1.053)	0.250
Perceived quality of care	1.257 (1.210, 1.306)	< 0.000			
Age in years					
25–44	1			1	
45–64	1.160 (1.060, 1.268)	0.001		1.156 (1.058, 1.264)	0.001
65+	1.305 (1.151, 1.480)	0.000		1.275 (1.123, 1.446)	< 0.000
Gender					
Male	1			1	
Female	0.974 (0.848, 1.118)	0.705		0.973 (0.847, 1.117)	0.695
Current marital status					
Unmarried	1			1	
Married	0.863 (0.727, 1.024)	0.092		0.870 (0.733, 1.034)	0.113
Attend formal education					
No	1			1	
Yes	0.951 (0.860, 1.052)	0.334		0.939 (0.848, 1.039)	0.220
Place of residence					
Rural	1			1	
Semi-urban	0.958 (0.848, 1.081)	0.485		0.986 (0.872, 1.115)	0.825
Household size	1.010 (0.985, 1.036)	0.441		1.012 (0.986, 1.038)	0.377
Wealth index					
Poor	1			1	
Middle	0.922 (0.837, 1.016)	0.101		0.932 (0.846, 1.027)	0.153
Rich	0.817 (0.710, 0.941)	0.005		0.825 (0.716, 0.950)	0.008
Insurance status					
Ex-member	1			1	
Active member	1.198 (1.056, 1.359)	0.005		1.199 (1.057, 1.360)	0.005
Chronic illness					
No	1			1	
Yes	1.188 (1.085, 1.302)	< 0.000		1.183 (1.080, 1.296)	< 0.000
Perceived health status					
Poor	1			1	
Moderate	0.760 (0.687, 0.842)	< 0.000		0.769 (0.694, 0.852)	< 0.000
Good	0.690 (0.616, 0.774)	< 0.000		0.692 (0.617, 0.777)	< 0.000

CI: Confidence Interval; IRR: Incidence Rate Ratio

Model II included the five quality of care dimensions while controlling for the confounding effect of covariates. Three quality dimensions, namely provider communication, information provision, and access to care, were found to be significantly correlated to the number of outpatient visits. The number of outpatient visits increases by a factor of 1.052 as the mean score of provider communication rises by one unit (95% CI: 1.012, 1.095; *p* = 0.011). For a one-point increase in the mean score of the information provision and access to care dimensions, the number of outpatient visits increases by a factor of 1.088 and 1.058, respectively (95% CI: 1.058, 1.120; *p* < 0.001 and 95% CI: 1.026, 1.091; *p* < 0.001).

Among the covariates, age of the household head, CHI membership status, wealth index, existence of chronic illness, and perceived health status were significantly associated with the number of outpatient visits, as shown in Model II. Outpatient visits are 1.275 and 1.156 times higher in households headed by individuals aged 65 + and 45 to 64 years, respectively, compared to those headed by individuals aged 25 to 44 years (95% CI: 1.123, 1.446; *p* < 0.001 and 95% CI: 1.058, 1.264; *p* = 0.001). Similarly, the number of outpatient visits for rich households is reduced by 17.5% compared to those who belong to poor households (95% CI: 0.716, 0.950; *p* = 0.008). Households that were active members of CHI at the time of the study had 1.199 times the number of outpatient visits as previous members (95% CI: 1.057, 1.360; *p* = 0.00).

With respect to health status, the number of outpatient visits among households that had a chronic illness in their family increased by 18.3% compared to those without a chronic illness (95% CI: 1.080, 1.296; *p* < 0.001). Furthermore, the number of outpatient visits among households that rated their health status as good and moderate was lower by 30.8% and 23.1%, respectively, compared to those who rated it as fair (95% CI: 0.617, 0.777; *p* < 0.001 and 95% CI: 0.694, 0.852; *p* < 0.001).

## Discussion

This study examined how the perception on quality of care relate to the number of outpatient visits in the nearby health centers among households. According to the findings, a typical household makes about 4.10 outpatient visits to nearby health centers per year. Health care utilization, as measured by the number of outpatient visits per household member, was 0.99 visits per person per year. This is lower than the findings of a previous study in Ethiopia, which reported outpatient visits of 1.77 per person per year [37]. This could be due to differences in measurement of the outcome variable. Outpatient visits in the previous study refers to the number of health facility visits made by a household for any type of health services, including curative, follow-up, and health promotion services, in any health facility during a one-month period preceding the study, whereas in the current study, it refers to the number of outpatient visits to a nearby health center made by a household for curative health services during the 12-month period prior to the study.

The findings demonstrated that the perception on the quality of outpatient service was a predictor of the number of annual outpatient visits. This is consistent with other studies which support the view that positive experience with healthcare service would prompt patients to revisit the service provider [13–15] and attend for scheduled appointments [16]. It was also documented that a greater number of problems related to the quality of primary care provisions, including issues related to access, continuity of care, provider communication and coordination, as perceived by users, was negatively associated with health care utilization [19]. A systematic review identified that perceived poor quality of care pushed patients away from the lower-level health facilities, because they did not trust primary level facilities to address their basic health needs [20]. It was also indicated that the better the perceived quality of care of a health facility, the more likely that facility being chosen [23, 38, 39], and the belief that the health system works well and only requires minor changes was associated with having a usual source of care [40]. Moreover high ratings of user-reported quality of care is a positive predictor of patients recommendation of the healthcare facility to a friend or family member [21].

As for the linkage between the number of outpatient visits and the different perceived quality of care dimensions, a significant association was found for the provider communication, information provision, and access to care dimensions. Previous work has indicated to the positive impact of the provider-patient interaction dimension of health care quality on patients’ loyalty [41]. The assurance dimension of perceived quality of care, which refers to care providers’ knowledge and courtesy, and their ability to inspire trust and confidence has a positive effect on use of outpatient services [42] as well as behavioral intentions of patients [14]. Likewise, the empathy dimension of perceived quality of care showed a significant association with the use of outpatient services [42]. This involves the attention given to clients by service providers, including ease of making relationships, good communication and understanding their needs. Effective provider communication is a fundamental clinical skill that facilitates the establishment of a relationship of trust between the health care provider and the patient, contributing to an increase in the prestige of the medical unit and the growing interest of patients in it [43].

Provision of information to patients has an important bearing on repeated visits of a health facility. Users’ perception on the quality dimension that related to physician description of illness, causes, and treatment plan has a positive effect on the outpatients’ choice of health facility [23]. A study reported a strong association between providers’ information provision and patient’s stated intent to return [44]. That means the caregivers showed an intent to return to the same facility if the provider told them the child’s illness, and the symptoms that would indicate a need for immediate return to the facility, discussed a return visit, and counselled them on feeding the child. Similarly, information and communication dimension, which refers to providing timely information to the clients, listening to their problems carefully and proper counselling by care providers has a positive influence on behavioural intention [45].

In support of the importance of communication and information provision dimensions, a study documented that patients’ recommendation of the physician to their family and friends was influenced by their perceptions of physicians’ communications, which include asking probing questions, listening to patients’ problems without interruptions, giving sufficient time to patients to explain their problems, clarifying their doubts and advising them on future course of action by doctors [46].

Access to care is another quality dimension that is associated with outpatient visits of health centers. This includes availability of essential medicines, reasonable waiting time, fair treatment of patients and friendly approach of facility assistants. In support if this finding, another study showed that increased waiting time decreases the probability of a health facility choice [47]. It is also documented that household’s healthcare utilization was positively and significantly associated with continuous availability of essential medicines [17, 48]. Moreover, limited medicines variety at lower-level health facilities cause patients to access higher levels [20].

Among the covariates, age of the household head, CHI membership status, wealth index, existence of chronic illness and perceived health status were significantly associated with the number of outpatient visits. Increased in the age of the household is associated with higher number of outpatient visits. This finding is consistent with the literature, which shows that older age, particularly being 65 or older, is associated with an increase in the number of outpatient visits [49, 50], first choice of primary health care facilities [51], and health care utilization [52, 53]. This could be because the occurrence of disease, particularly chronic illnesses, increases with age, resulting in a greater need for healthcare.

This study demonstrated that the number of outpatient visits of households who belong to the poor wealth class was higher than that of the rich class. This finding mirrors prior study which showed that higher and middle wealth class households were less likely to seek outpatient services from primary health centers compared to the lower class [54]. With respect to the choice of health facility, higher income is also inversely related to the use of primary health care facilities [20, 51], as the better off families may have the demand to use better equipped and advanced health facilities. In contrary, it is documented that increased income is associated with higher probability of using health care, as it removes the financial barrier of access to care [52, 55, 56]. In the current study, the low number of outpatient visits in health centers among the rich class might not indicate low utilization of health care, rather it might be because of their preference to visit higher level or private health facilities. The poor might not have the financial means to seek care beyond the nearby health centers, which are relatively less costly. This is supported by the evidence that patients who can afford the cost of care often choose access at higher levels [20]. Similarly, an increase in hospital price cause patients to choose primary health care facilities for outpatient visits [57].

Households who were active members of CHI had a higher number of outpatient visits compared to those who quitted their membership. Findings of this study echo earlier evidence which showed that having an insurance plan is linked to an increased in health care utilization [37, 48, 53, 58–60]. While removal of financial barriers to health care use is a possible explanation for the observed result, another is the presence of moral hazard behavior due to having health insurance. In the latter situation, people with insurance coverage tend to use more outpatient services because they know that the scheme will bear the medical bills [61]. Another plausible explanation is the gatekeeping effect of CHI membership. In the study area, CHI members have to follow the referral path in order to receive the scheme’s benefit packages. Member households are required to first visit the designated health centers and need to get referrals so as to receive health care at the next higher level health facility i.e. public hospitals [62]. As a result, their healthcare utilization is limited to the lower-level health facilities until they are referred to the higher level. In support of this view, a study revealed that patients with gatekeepers were more likely to choose community health centers first when seeking care, compared with patients having freedom of choice to seek medical care at any place [63].

The existence of chronic illness within the household was also linked to an increase in the number of outpatient visits. This is corroborated by the literature that having at least one chronic disease increased the number of outpatient visits [50, 52, 58], and promote first choice of PHC facilities [51]. This is because people with chronic illnesses have to visit health care facilities frequently for follow up cares that can be provided by PHC facilities.

The household head’s subjective assessment of the health status of the family has also an important bearing on the number of outpatient visits. Those who rated the health of the household as poor had higher number of outpatient visits compared to those who rate it as moderate and good. This is consistent with the existing evidence which showed that perceived poor health status is linked to more outpatient care utilization [49, 50, 52, 56]. This may be true for people who perceive their health as poor to understand and value the need to seek healthcare, and to visit health facilities when the need arises.

The findings of this study will be an essential input for quality improvement endeavors as well as addressing challenges in efforts to attain universal health coverage. It provides a valuable lesson for Ethiopia and other low-income countries about the essence of enhancing the quality of care in order to leverage primary health care units while reducing the strain on higher-level health facilities. Despite the study gives an important lesson to healthcare managers and other relevant stakeholders, it is not without limitations. The study might be prone to recall bias in assessing the number of annual outpatient visits made by the household. Second, response bias is another possibility, as the head of the household might not have the full information on the use of outpatient services by all family members. Third, households that had not visited health centers for outpatient services in the 12 months period prior to the study were excluded to minimize recall bias. This non-utilization might be due to prior negative experience with the service providers. Fourth, the study fails to include some important covariates like the occurrence of acute illness in the last one year and its severity, which might confound the association between quality of care and outpatient visits. Finally, the study would not be immune to interviewer bias, despite efforts to minimize it by training data collectors on the purpose of the study, and how to use the data collection tool.

## Conclusions

The current research showed that clients’ perception on the quality of care delivered at health centers is vital to attracting patients to the same facility for outpatient visits. Subscales of the perceived quality of care, particularly provider communication, information provision and access to care are strong predictors of the number of outpatient visits, showing the need to addressing issues related to quality of care. Unless patients are receiving a better quality of care, they might distrust and develop a negative attitude towards the health facility. Hence, strong efforts are required to improve the quality of care that rely on perception of clients with a special focus on improving communication skills of health care providers and removing facility level access barriers so as to boost clients’ interest to utilize primary health care facilities.

### Electronic supplementary material

Below is the link to the electronic supplementary material.

Supplementary Material 1

## Data Availability

The datasets used and/or analyzed during the current study are available from the corresponding author on reasonable request.

## Abbreviations

AIC: Akaike Information Criterion
BIC: Bayesian Information Criterion
CHI: Community Health Insurance
DIC: Deviance Information Criterion
IRR: Incidence Rate Ratio
ZTNB: Zero-Truncated Negative Binomial
ZTP: Zero-Truncated Poisson
