# Supplementary material for: The role of perceived quality of care on outpatient visits to health centers in two rural districts of northeast Ethiopia: a community-based, cross-sectional study
Source: BMC Health Serv Res. 2024 May 10;24:614. doi: 10.1186/s12913-024-11091-z (PMC11084123; doi:10.1186/s12913-024-11091-z)
Supplement: Supplementary file 1 — Supplementary Material 1 [file 12913_2024_11091_MOESM1_ESM.pdf]

Questionnaire- English version

Title: The role of perceived quality of care on outpatient visits to health centers in two rural districts of northeast Ethiopia: a community-based, cross-sectional study

SECTION I: Household and Respondent's Background Characteristics

A. Demographic and socio-economic characteristics of respondents				
**S.N**	**Questions and filter**	**Response categories & coding**		**Skip**
101.	Participant's ID	________________		
102.	What is your age?	______________ Years		
103.	What is your sex?	Male ....<br>Female	1<br>2	
104.	What is your current marital status?	Single ....<br>Married ....<br>Divorced ....<br>Widowed	1<br>2<br>3<br>4	
105.	Place of residence	Rural ....<br>Semi-urban	1<br>2	
106.	Have you ever attended school?	Yes ....<br>No	1<br>2	 → 108
107.	What is the highest level of education you have completed?	Grade_______________		
108.	What is your current occupation?	Farmer ....<br>Merchant ....<br>Daily worker.........<br>Other (Specify)......	1<br>2<br>3<br>4	
109.	How would you rate the overall health of the household?	Poor ....<br>Fair...................<br>Good ....<br>Very good ....<br>Excellent	1<br>2<br>3<br>4<br>5	
110.	When was the last time that any member of the household had a consultation with a health professional? In the last	30 days<br>1 to 3 months<br>3 to 6 months<br>6 to 12 months<br>More than 12 months	1<br>2<br>3<br>4<br>5	
111.	How many times any of a household member visited the outpatient unit of nearby health center in the past 12 months?	_________________ visits		
112.	What is your households CBHI insurance status now	Active member...........<br>Ex-member	1<br>2	
113.	Your CBHI card number	________________		
114.	Year of enrolment to CBHI (see CBHI ID)	_________________E.C		
115.	Have you dropped out of CBHI after enrollment?	Yes........................<br>No	1<br>2	 → 117
116.	At what year did you drop out of CBHI? (see CBHI ID)	_________________E.C		
117.	Have you re-enrolled in CBHI after dropping out of the scheme?	Yes........................<br>No	1<br>2	 → 118
118.	When did you re-enrolled (see CBHI ID)	_____________year(E.C)		
119.	How many persons live in this household	______________ Persons		
120.	Is/was there a member of the family with clinically diagnosed chronic illness?	Yes ....<br>No	1<br>2	

**B. Household wealth Characteristics**			
**No.**	**Questions and filter**	**Response categories & coding**	**skip**
121.	What is the main source of **drinking water** for members of your household?	Piped Water .... 1<br>Tube well/borehole .... 2<br>**Dug Well**<br>Protected well .... 3<br>Unprotected well .... 4<br>**Water from Spring**<br>Protected spring.................... 5<br>Unprotected spring.............. 6<br>Surface water [1].......................... 7<br>Other (Specify) _______________ 8	
122.	What kind of toilet facility do members of your household usually use?	Ventilated improved pit latrine .... 1<br>Pit latrine with slab........................ 2<br>Pit latrine without slab/open pit...... 3<br>No facility/bush/field 4	4 → 124
123.	Do you share this toilet facility with other households?	Yes .... 1<br>No 2	
124.	What type of fuel does your household mainly use for cooking?<br>***[Multiple option possible]***	Electricity .... 1<br>Kerosene .... 2<br>Charcoal .... 3<br>Wood .... 4<br>Shrubs/agricultural crop............ 5<br>Animal dung .... 6<br>Others (Specify)____________ 7	
125.	What type of fuel does your household mainly use for source of light?<br>***[Multiple option possible]***	Electricity .... 1<br>Lamp .... 2<br>Kerosene lamp .... 3<br>Solar light .... 4<br>Wood .... 5<br>Others (Specify)____________ 6	
126.	How many rooms are used for sleeping?	________________ Rooms	
127.	How many of the following animals does this household own?<br>*(If none, record '00')*	a. Milk cows, oxen or bulls...... ____<br>b. Other cattle..................... ____<br>c. Horses/donkeys/mules .... ____<br>d. Camels .... ____<br>e. Goats/Sheep.................... ____<br>f. Chickens or other poultry.... ____<br>g. Beehives .... ____	
128.	Does any member of this household own any agricultural land?	Yes.................................... 1<br>No 2	2 → 130
129.	How many hectares of agricultural land do members of this household own?	Sq. meter_________ or “Timad” _________	

[1] River/lake/dam/pond/ stream/canal/ irrigation channel etc.

130.	Does your household have:	Yes No<br>a. Electricity .... 1 2<br>b. Radio .... 1 2<br>c. Television .... 1 2<br>d. Telephone (non-mobile) 1 2<br>e. Computer .... 1 2<br>f. Refrigerator .... 1 2<br>g. Table .... 1 2<br>h. Solar light .... 1 2<br>i. Bed with cotton/mattress. 1 2<br>j. Lamp 1 2	
131.	Does any member of this household own?	Yes No<br>a. Watch .... 1 2<br>b. Mobile telephone .... 1 2<br>c. Bicycle .... 1 2<br>d. Motorcycle or scooter ... 1 2<br>e. Animal-drawn cart .... 1 2<br>f. Car or Truck .... 1 2<br>g. Bajaj 1 2	
132.	Does any member of this household have a bank account?	Yes .... 1<br>No 2	
133.	**Main** material of the **FLOOR** of the dwelling (observation)	**Natural Floor** .... 1<br>Earth/sand/dung<br>**Rudimentary Floor** .... 2<br>Wood planks/Palm/bamboo<br>**Finished Floor** .... 3<br>Parquet or polished wood<br>Vinyl or asphalt strips<br>Ceramic tiles<br>Cement<br>Carpet	
134.	**Main** material of the **ROOF** of the dwelling (observation)	**Natural Roofing**.................. 1<br>No roof/thatch/mud/sod<br>**Rudimentary Roofing** .... 2<br>Rustic mat/palm/bamboo/wood planks/wood cardboard<br>**Finished Roofing** .... 3<br>Metal/corrugated iron /wood /cement /ceramic tiles/roofing shingles	
135.	**Main** material of the exterior **WALL** of the dwelling (observation)	**Natural walls** .... 1<br>No walls/Cane/palm/trunks/dirt<br>**Rudimentary** .... 2<br>Bamboo with mud/stone with mud/uncovered adobe/plywood/ cardboard/reused wood<br>**Finished walls** .... 3<br>Cement/stone with lime/cement/ bricks/cement blocks/covered adobe/wood planks/shingles	

SECTION III: Perceived health care quality

The following questions are concerning the health facility which is the usual source of health care for your family members. Respond to the questions based on what you have observed or experienced on the health service while you seek care or accompanying your family members.

No.	Health care quality measurement items	Strongly disagree	Disagree	Indifference	Agree	Strongly agree
301.	The health facility environment is clean (i.e., OPD rooms, toilets, waiting room etc.)	1 ☐	2 ☐	3 ☐	4 ☐	5 ☐
302.	The waiting room has enough space and seating	1 ☐	2 ☐	3 ☐	4 ☐	5 ☐
303.	Patients do not wait long in the health center to receive treatment	1 ☐	2 ☐	3 ☐	4 ☐	5 ☐
304.	The health facility serves all patients fairly	1 ☐	2 ☐	3 ☐	4 ☐	5 ☐
305.	Health care providers treated you with courtesy and respect	1 ☐	2 ☐	3 ☐	4 ☐	5 ☐
306.	Health care providers spent sufficient time to examine and discuss your health problem	1 ☐	2 ☐	3 ☐	4 ☐	5 ☐
307.	Health care providers actively ask questions to better understand your situation	1 ☐	2 ☐	3 ☐	4 ☐	5 ☐
308.	Health care providers listened to you carefully what you had to say	1 ☐	2 ☐	3 ☐	4 ☐	5 ☐
309.	Health professionals perform the necessary physical examinations	1 ☐	2 ☐	3 ☐	4 ☐	5 ☐
310.	The necessary Lab. and other tests are done	1 ☐	2 ☐	3 ☐	4 ☐	5 ☐
311.	Health care providers make good diagnosis	1 ☐	2 ☐	3 ☐	4 ☐	5 ☐
312.	Health professionals prescribe the appropriate medication for the patient	1 ☐	2 ☐	3 ☐	4 ☐	5 ☐
313.	All prescribed drugs are available on the spot	1 ☐	2 ☐	3 ☐	4 ☐	5 ☐
314.	Treatment is effective for recovery and cure	1 ☐	2 ☐	3 ☐	4 ☐	5 ☐
315.	You have confidence and trust in the health care providers examining and treating patients	1 ☐	2 ☐	3 ☐	4 ☐	5 ☐
316.	Health care providers treat you with care and understand your concerns	1 ☐	2 ☐	3 ☐	4 ☐	5 ☐
317.	Health care providers explain things in a way you could understand	1 ☐	2 ☐	3 ☐	4 ☐	5 ☐
318.	Health care providers clearly explained to you the results of the tests and examination	1 ☐	2 ☐	3 ☐	4 ☐	5 ☐
319.	Health care providers explained the use and side effects of medications you were to take at home in a way you could understand	1 ☐	2 ☐	3 ☐	4 ☐	5 ☐
320.	Facility assistants are friendly and helpful to patients	1 ☐	2 ☐	3 ☐	4 ☐	5 ☐
321.	Overall, how would you rate the health services you received?	1 ☐ Poor	2 ☐ Fair	3 ☐ Good	4 ☐ Very Good	5 ☐ Excellent
